# Supplementary material for: Whole genome analysis identifies the association of TP53 genomic deletions with lower survival in Stage III colorectal cancer
Source: Sci Rep. 2020 Mar 19;10:5009. doi: 10.1038/s41598-020-61643-6 (PMC7081316; doi:10.1038/s41598-020-61643-6)
Supplement: Supplementary file 3 — Supplementary Information3. [file 41598_2020_61643_MOESM3_ESM.pdf]

SUPPLEMENTARY MATERIALS

Whole genome analysis identifies the association of *TP53* genomic deletions with lower survival in Stage III colorectal cancer

AUTHORS

Li C Xia[1], Paul Van Hummelen[1], Matthew Kubit[2], HoJoon Lee[1], John M Bell[2], Susan M. Grimes[2], Christina Wood-Bouwens[1], Stephanie U. Greer[1], Tyler Barker[3], Derrick S Haslem[3], James Ford[1], Gail Fulde[3], Hanlee P Ji[1,2,*], Lincoln D Nadauld[3,*]

Affiliations:

[1]Division of Oncology, Department of Medicine, Stanford University School of Medicine, Stanford, CA, United States.

[2]Stanford Genome Technology Center, Stanford University, Palo Alto, CA, United States.

[3]Precision Genomics Program, Intermountain Healthcare, Saint George, UT, United States.

*Corresponding authors

Hanlee P. Ji

genomics_ji@stanford.edu

Lincoln D. Nadauld

Lincoln.Nadauld@imail.org

Running title: whole genome analysis of Stage III colorectal cancer for prognostic genomic markers

SUPPLEMENTARY FIGURES

Supplementary Figure S1. A shoulder-to-shoulder view of the segmentation tracks estimated from the low-coverage and high-coverage WGS platforms from the same sample. Y-axis was drawn in the same scale. This suggests for this high purity tumor sample, the low-coverage WGS-derived segments are highly accordant to high-coverage WGS's.

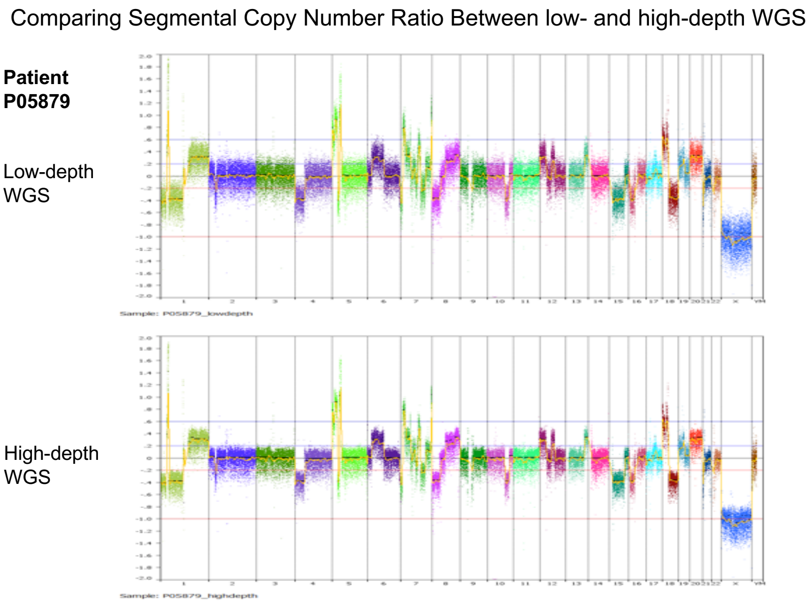

Supplementary Figure S2. A shoulder-to-shoulder view of the segmentation tracks estimated from the low-coverage WGS and WES platforms from the same sample. Y-axis was drawn in the same scale. This suggests WES-derived segments are significantly more variable as compared to low-coverage WGS.

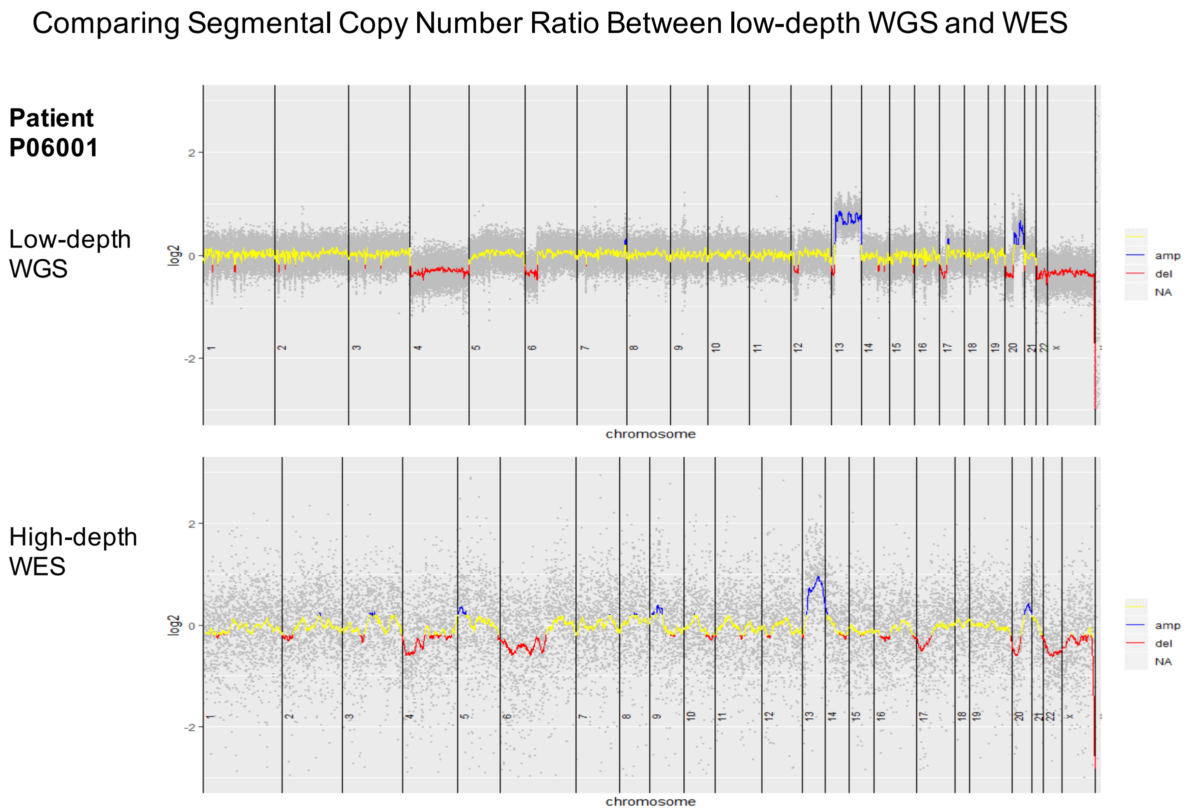

Supplementary Figure S3. Arm-level chr17p loss predicts for poorer survival in Stage III CRC. The Kaplan–Meier plots as stratified by patients' status of carrying the chr17p arm loss (SCNA_CHR_ARM_17p_del=1 for yes, otherwise 0), for which 11 patients of the Stage III CRC discovery cohort who clinically tested either as MSI-high by PCR or positive by MMR-IHC were excluded.

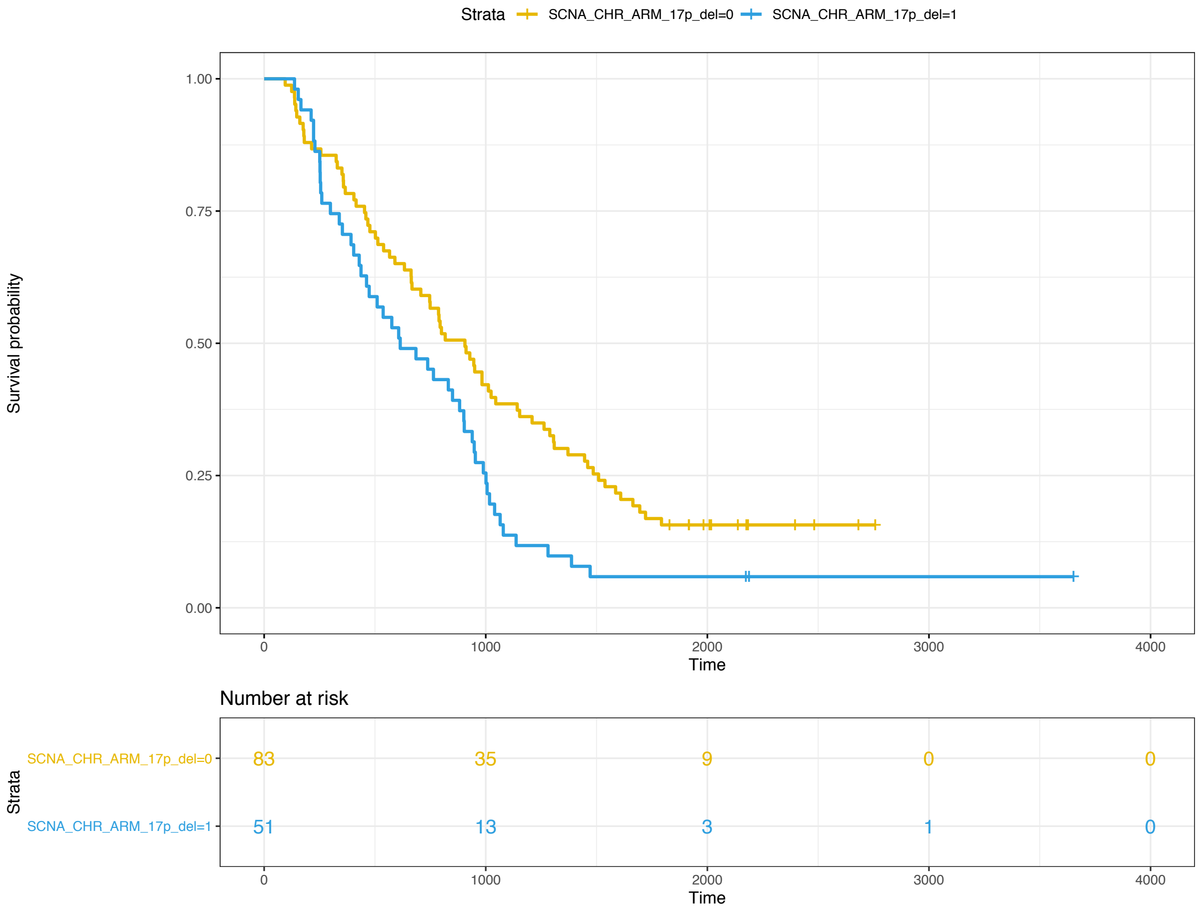

Supplementary Figure S4. mRNA expression difference between patients with and without TP53 deletion in the TCGA cohort. This suggests TP53 deletion is associated with significantly reduced mRNA expression level of TP53.

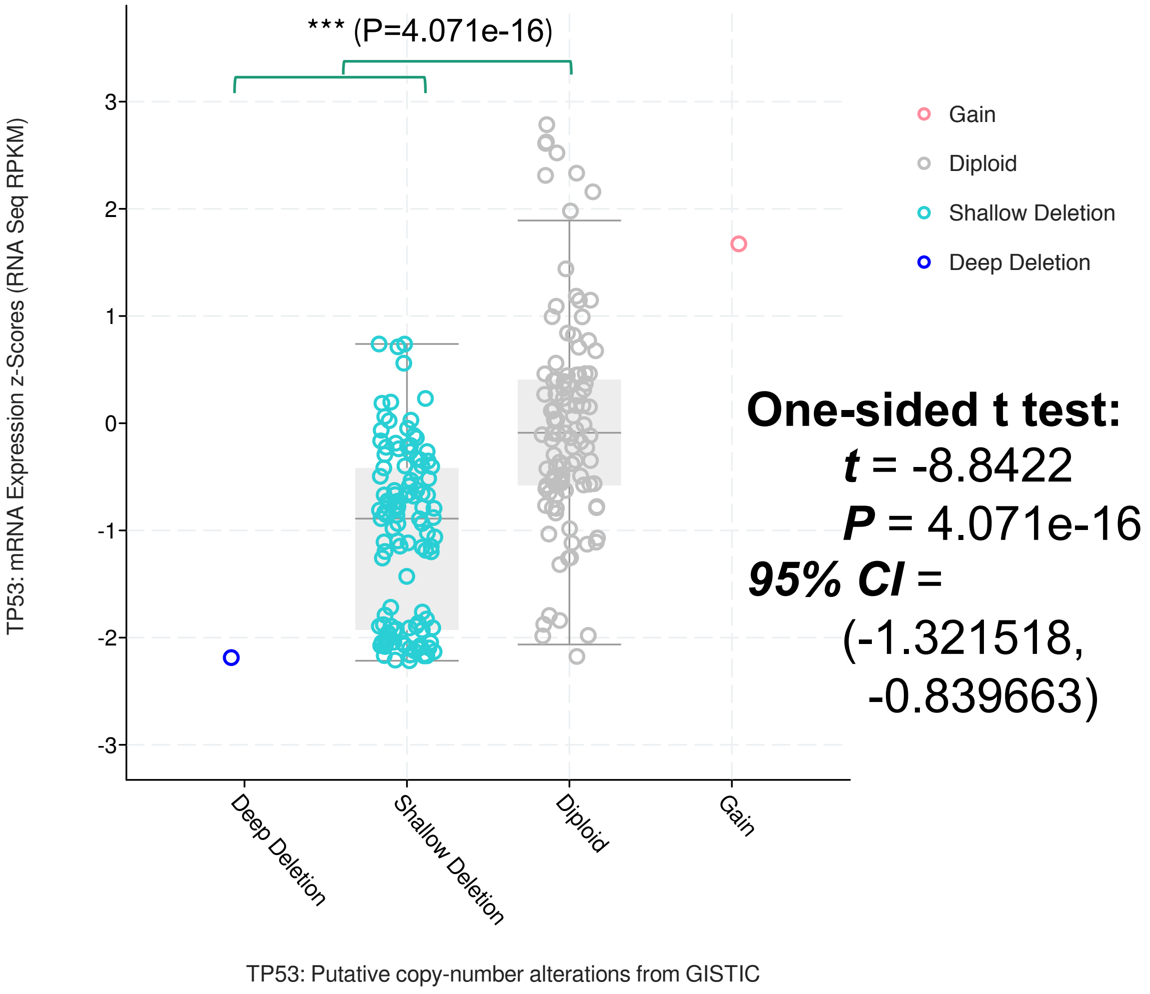

Supplementary Figure S5. Estimated per-sample cost of low-depth (2-4x) WGS profiling of CNAs. We projected the per-sample costs to future years using a log-linear model based on the first available year of the most economical platform.

	Incremental cost of low-coverage (2-4x) WGS profiling of CNAs.				
**Cost/Sample ($)**		**Sample Pool Size**			
		~10	~100	~200	~500
**Analysis Platform**	**Available Year**				
SNP-array 6.0	2018 - 2025	200	200	200	200
MiSeq	2011 - 2019	658 - 1,316	658 - 1,316	658 - 1,316	658 - 1,316
NextSeq 500	2014 - 2019	480 - 960	420 - 840	420 - 840	420 - 840
HiSeq 2500	2012 - 2019	n. a.	196 - 392	196 - 392	196 - 392
HiSeq 4000	2015 - 2019	n. a.	127 - 254	127 - 254	127 - 254
HiSeq X	2014 - 2019	n. a.	n. a.	86 - 172	86 - 172
NovaSeq 6000	2017 - 2019	n. a.	120 - 240	94 - 188	77 - 144
Projected Cost*	2022	480 - 960	104 - 208	44 - 88	22 - 44
Projected Cost*	2025	480 - 960	95 - 190	28 - 56	20 - 40

(orange)	per sample cost comparable to microarray
(green)	per sample cost lower than microarray
n.a.	platform not applicable given sample pool size
